# Development of in vitro resistance to fluoroquinolones in *Pseudomonas aeruginosa*

**DOI:** 10.1186/s13756-020-00793-8

**Published:** 2020-08-05

**Authors:** Lei Zhao, Shiqi Wang, Xiaobing Li, Xiaojing He, Lingyan Jian

**Affiliations:** 1 Department of Pharmacy, Shengjing Hospital of China Medical University, NO. 36, Sanhao Street, Heping District, Shenyang, 110004 China; 2 School of Pharmacy, China Medical University, NO. 77, Puhe Street, Shenbei New District, Shenyang, 110001 China

**Keywords:** Fluoroquinolone, *Pseudomonas aeruginosa*, Efflux pumps, Ciprofloxacin, Levofloxacin

## Abstract

Fluoroquinolone resistance in *Pseudomonas aeruginosa* typically arises through site-specific mutations and overexpression of efflux pumps. In this study, we investigated the dynamics of different resistance mechanisms in *P. aeruginosa* populations that have evolved under fluoroquinolone pressure, as well as the interactions between these mechanisms in evolutionary trajectories. Bacteria of strain ATCC27853 were selected under different concentrations of ciprofloxacin and levofloxacin for six parallel lineages, followed by amplification of four target genes in the quinolone-resistance determining region (QRDR) and Sanger sequencing to identify the mutations. The expression of four efflux pump proteins was evaluated by real-time polymerase chain reaction using the relative quantitation method, with the ATCC27853 strain used as a control. We found that ciprofloxacin killed *P. aeruginosa* sooner than did levofloxacin. Further, we identified five different mutations in three subunits of QRDRs, with *gyrA* as the main mutated gene associated with conferring fluoroquinolone resistance. Additionally, we found a larger number of mutations appearing at 2 mg/L and 4 mg/L of ciprofloxacin and levofloxacin, respectively. Moreover, we identified the main efflux pump being expressed as MexCD-OprJ, with initial overexpression observed at 0.25 mg/L and 0.5 mg/L of ciprofloxacin and levofloxacin, respectively. These results demonstrated *gyrA*^83^ mutation and MexCD-OprJ overexpression as the primary mechanism conferring ciprofloxacin and levofloxacin resistance in *P. aeruginosa*. In addition, we also show that ciprofloxacin exhibited a stronger ability to kill the bacteria while potentially rendering it more susceptible to resistance.

## Background

*Pseudomonas aeruginosa* is a Gram-negative, opportunistic human pathogen and is considered to be one of the main pathogens associated with nosocomial infections. *P. aeruginosa* is the key agent responsible for cystic fibrosis in lung infections [1], and infectious lesions caused by this bacteria can result in blood-borne transmission, bacteremia, and sepsis. Additionally, severe *P. aeruginosa* infections in burn patients can lead to death. Fluoroquinolones (FQs), which have favorable pharmacokinetic/pharmacodynamic properties, are a major class of antibiotics used to treat *P. aeruginosa* infections, with the most commonly used FQs for the treatment being ciprofloxacin and levofloxacin. *P. aeruginosa* exhibits a large variety of available resistance mechanisms, which can act in combination and render even the most potent drugs useless [2].

The most prevalent mechanisms contributing to FQ resistance in *P. aeruginosa* involve mutations in quinolone-resistance determining regions (QRDRs), such as *gyrA* and *gyrB* in DNA gyrase and *parC* and *parE* in topoisomerase IV, along with overexpression of resistance–nodulation–division efflux pumps (i.e., MexAB-OprM, MexCD-OprJ, MexEF-OprN, and MexXY-OprM) [3]. Epidemiological analyses indicate that high-level resistance to FQ requires QRDR mutations in at least two genes [4], and that overexpression of the MexEF-OprN efflux pump represents a major mechanism by which *P. aeruginosa* can acquire higher FQ-resistance levels [5].

Microbial resistance is an evolutionary response to antibiotic pressure, and evolutionary steps result in alteration of drug susceptibility to clinical resistance. Microbial evolution experiments comprise a powerful approach to examining pathogen adaptation to antibiotics during the evolutionary process in real-time and under highly controlled laboratory conditions [4]. Recent studies have established causal links between antibiotic deployment therapies and the course/timing of mutations, the cost of resistance, and the likelihood of developing compensating mutations [6]. The main limitation of using experimental animal models to investigate resistance mechanisms is the risk of spreading the plasmid to the surroundings. Additionally, a combination of drugs is usually used in the therapy, which may interfere with the experiments investigating the mechanisms associated with FQ resistance in *P. aeruginosa*. Therefore, the purpose of this study was to develop an understanding of chromosome-mediated resistance using an in vitro*-*selection model to block communication between *P. aeruginosa* and the environment.

## Materials and methods

### Bacterial strains, growth conditions, and minimum inhibitory concentration (MIC) determination

For in vitro resistance-selection studies, *P. aeruginosa* ATCC27853 was obtained from the American Type Culture Collection (Manassas, VA, USA) and grown in Mueller–Hinton broth (MHB) or on Mueller–Hinton agar plates for 18 h to 24 h in a 5% CO_2_ atmosphere at 37 °C. Antibiotic susceptibility to ciprofloxacin and levofloxacin was determined by the agar dilution method according to Clinical & Laboratory Standards Institute (CLSI)-approved standards. MIC determination was performed in triplicate, using the same experimental conditions for all lineages.

### Selection of FQ-resistant *P. aeruginosa* populations by serial passages

Six independent lineages (L1–L6) for ciprofloxacin and levofloxacin were established and propagated by serial passages with increasing concentrations of antibiotics. Selection of 200 μL of bacteria was performed in flasks containing 20 mL MHB supplemented with various concentration gradients of ciprofloxacin and levofloxacin, starting with an initial concentration of 0.5MIC. Bacteria were sub-cultured for 24 h at 37 °C in the flasks, and resistant bacteria were serially transferred to new flasks containing fresh medium with 2-fold serial antibiotic concentrations. The procedure was repeated until growth was markedly inhibited. At each selection passage, a sample of the bacterial population from each passage was collected with 15% sterilized glycerol and frozen in cryotubes at − 80 °C.

### Bacterial DNA extraction

Bacterial DNA extracts used as templates in this study were prepared by the heat-boiling method. Bacterial suspensions were centrifuged at 12,000 rpm for 10 min to collect bacteria, which were resuspended in 500 μL of sterilized water and incubated at 100 °C for 10 min. The cooling mixture was then centrifuged at 12,000 rpm for 5 min, and supernatants were collected and stored at − 20 °C until use.

### Gene amplification and sanger sequencing of DNA gyrase and topoisomerase IV

The QRDRs of the *gyrA*, *gyrB*, *parC*, and *parE* genes of serially passaged *P. aeruginosa* populations were amplified by polymerase chain reaction (PCR) using gene-specific primers (Supple. Table 1). To amplify each gene, 1 μg of the DNA template was subjected to PCR amplification in a 30 μL reaction mixture containing 12 μM of each forward and reverse primer, 7.5 μM of dNTPs, 3 μL 10× Ex *Taq* buffer (with MgCl_2_), and 1.5 U of Ex *Taq* DNA polymerase (TaKaRa, Shiga, Japan). PCR amplification was performed under the following conditions: for *gyrA* and *gyrB* amplification, denaturation was carried at 94 °C for 5 min, followed by 36 cycles of amplification each at 94 °C for 30 s, 58 °C for 30 s and 72 °C for 30 s, and then a final extension at 72 °C for 7 min; 2) for *parC* amplification, denaturation was carried at 94 °C for 5 min, followed by 40 cycles of amplification each at 94 °C for 30 s, 60 °C for 30 s*,* and 72 °C for 30 s, and then a final extension at 72 °C for 7 min; and 3) for *parE* amplification, denaturation was carried at 94 °C for 5 min, followed by 36 cycles of amplification each at 94 °C for 30 s, 70 °C for 30 s*,* and 72 °C for 30 s, and then a final extension at 72 °C for 7 min. Amplified PCR products of all genes were visualized on 2% agarose gels containing GoodView (Beijing SBS Genetech Co., Beijing, China) with a DNA marker (TaKaRa) under ultraviolet light. Amplified PCR products were sent to the Beijing Genomics Institute for Sanger sequencing using the above-mentioned PCR primers. Nucleotide sequences of genes were compared with corresponding reference sequences of *P. aeruginosa* ATCC27853.

### Evaluation of the expression of efflux pump genes

Expression levels of *mexA*, *mexC*, *mexE*, *mexX*, and the housekeeping gene *rpoD* were determined by quantitative real-time reverse transcription (qRT)-PCR. RNA was isolated using RNAiso reagent (TaKaRa), and cDNA was synthesized using the PrimeScript RT reagent kit with gDNA Eraser (TaKaRa), according to manufacturer’s instructions. qRT-PCR was performed in duplicate, in 20 μL volume with 100 ng RNA and primer concentration of 0.4 μM, on a QuantStudio DX Flex system (Applied Biosystems, Foster City, CA, USA) using SYBR Premix Ex Taq (TaKaRa). The primers for qRT-PCR are listed in Supple. Table 2. Gene expression was determined using the ΔΔCt method and a standard curve to measure PCR efficiency. All results were normalized to the expression of the housekeeping gene *rpoD* and calibrated relative to expression in *P. aeruginosa* ATCC27853.

## Results

### MIC determination and selection of FQ-resistant *P. aeruginosa* populations

The MICs of both ciprofloxacin and levofloxacin in *P. aeruginosa* ATCC27853 were 0.5 mg/L, which is within the ranges defined for ciprofloxacin (0–1 mg/L) and levofloxacin (0–2 mg/L), according to the 2015 CLSI. Six independent lineages were propagated from *P. aeruginosa* ATCC27853 by serial passages in liquid medium with increasing concentrations of ciprofloxacin and levofloxacin. We found that for different passages of six lineages (L1–L6) selected in case of ciprofloxacin treatment, L5 and L6 died at 4 mg/L (8MIC), whereas L2 and L3 were killed at 8 mg/L (16MIC). Additionally, we observed that L1 and L4 were significantly suppressed at 32 mg/L (64MIC), which was thus used as the end point for ciprofloxacin selection. In case of levofloxacin treatment, L2 was inhibited at 64 mg/L (128MIC) and L6 at 32 mg/L (64MIC), whereas L5 was killed at 32 mg/L (64MIC). We thus defined 32 mg/L (64MIC) as the end point for levofloxacin selection. Each lineage demonstrated eight independent passages (P1–P8) capable of survival at different levofloxacin concentrations (0.5–64MIC), which suggested that *P. aeruginosa* was more susceptible to ciprofloxacin than to levofloxacin.

### Determination of DNA gyrase and topoisomerase IV sequences

We then sequenced the QRDRs of *gyrA*, *gyrB*, *parC*, and *parE* in each passage of the six different lineages of resistant *P. aeruginosa*. We identified four different mutations in ciprofloxacin-resistant lineages (Table 1), with three mutations in *gyrA*, whereas the fourth mutation was the same as that identified in the experiment with levofloxacin (*gyrB*^466^). The three mutations in *gyrA* were *gyrA*^83^ (the same as that found in passages selected from levofloxacin), *gyrA*^87^ (in the first position of codon 87, where aspartic acid was replaced by asparagine [D87N; GAC → AAC]), and *gyrA*^87*^ (in the second position of codon 87, where aspartic acid was replaced by glycine [D87G; GAC → GGC]). Interestingly, we found that *gyrA*^83^ prolonged *P. aeruginosa* survival under ciprofloxacin pressure relative to that observed in lineages with only *gyrA*^87^ mutations. We found different results involving levofloxacin pressure (Table 2), with three different mutations found in different samples: *gyrA*^83^ (in the second position of codon 83, where threonine was replaced by isoleucine [T83I; ACC → ATC]); *gyrB*^466^ (in the second position of codon 466, where serine was replaced by phenylalanine [S466F; TCC → TTC]); and *parE*^457^ (in the first position of codon 457, where serine was replaced by cysteine [S457C; AGC → TGC]).

**Table 1:** Mutations in QRDR genes in evolved, resistant *P. aeruginosa*, selected by ciprofloxacin

Passage	Lineage1	Lineage2	Lineage3	Lineage4	Lineage5	Lineage6
0. (0MIC)	–	–	–	–	–	–
1. (1/2MIC)	–	–	–	–	–	–
2. (1MIC)	–	A87 + B	A87	–	–	–
3. (2MIC)	A83	A87 + B	A87	–	–	–
4. (4MIC)	A83	A87 + B	A87	A83	–	A87*
5. (8MIC)	A83	A87 + B	A87	A83	–	A87*
6. (16MIC)	A83	A87	A87 + B	A83	/	/
7. (32MIC)	A83	/	/	A83	/	/
8. (64MIC)	A83	/	/	A83	/	/

A83: *gyrA*^83^ (T83I; ACC → ATC); A87: *gyrA*^87^ (D87N; GAC → AAC); A87*: *gyrA*^87^ (D87G; GAC → GGC); B: *gyrB*^466^ (S466F; TCC → TTC)

**Table 2:** Mutations in QRDR genes in evolved, resistant *P. aeruginosa,* selected by levofloxacin

Passage	Lineage1	Lineage2	Lineage3	Lineage4	Lineage5	Lineage6
0. (0MIC)	–	–	–	–	–	–
1. (1/2MIC)	–	–	–	–	–	–
2. (1MIC)	A	–	–	A	–	–
3. (2MIC)	A	–	–	A	B	–
4. (4MIC)	A	–	–	A	B	–
5. (8MIC)	A + E	A	A + B	A	B	B
6. (16MIC)	A + E	A	A + B	A	B	B
7. (32MIC)	A + E	A	A	A	B	A + B
8. (64MIC)	A + E	A	A	A	B	A

A: *gyrA*^83^ (T83I; ACC → ATC); B: *gyrB*^466^ (S466F; TCC → TTC); E: *parE*^457^ (S457C; AGC → TGC)

We found that the *gyrA* mutations (*gyrA*^83^*, gyrA*^87^*,* and *gyrA*^87*^) were the most important mutations for *P. aeruginosa* resistance to ciprofloxacin and levofloxacin, whereas the *gyrB*^466^ and *parE*^457^ mutations were found together with the *gyrA* mutations. Additionally, we found that the *gyrB*^466^ mutation disappeared at higher MIC concentrations. Moreover, during levofloxacin selection, we identified a large number of mutations at 4 mg/L (8MIC) as compared with 2 mg/L (4MIC) in case of ciprofloxacin. This suggested that ciprofloxacin exhibited stronger selection for resistant *P. aeruginosa*.

### Evaluation of the expression of efflux pump genes

**Fig. 1:**
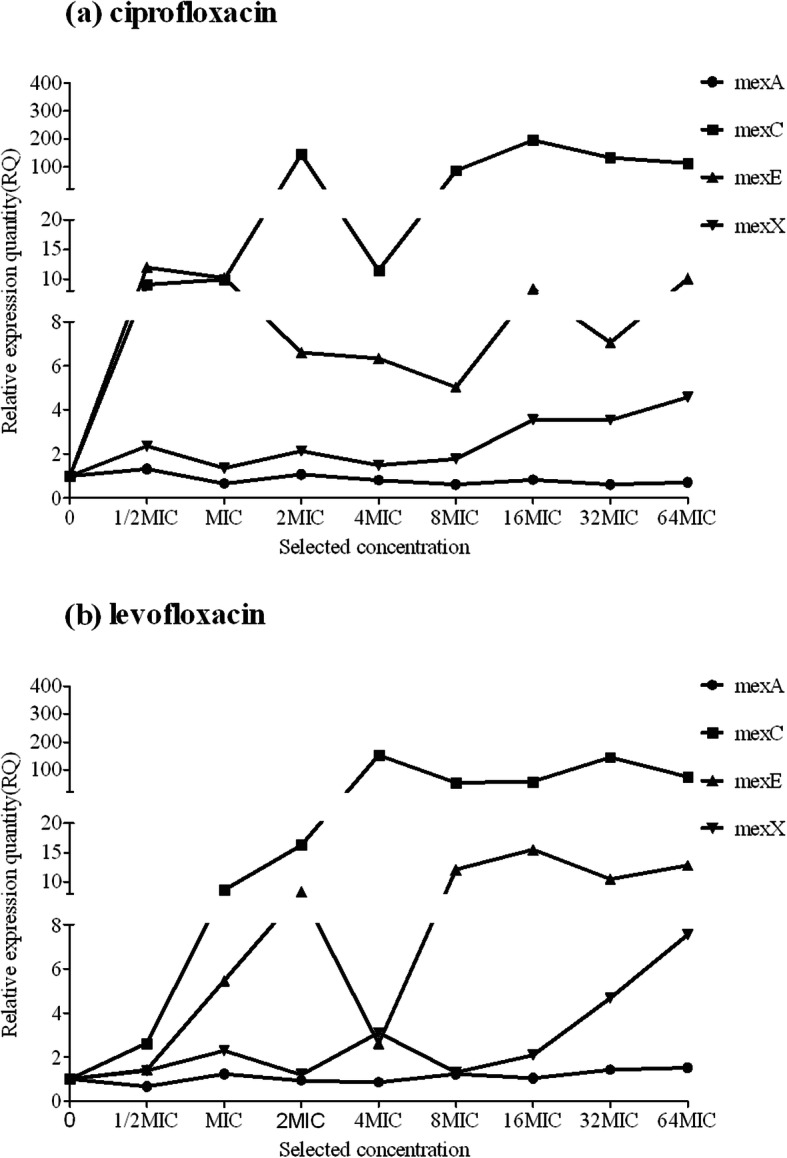
Relative expression of efflux pump genes in evolved, resistant *P. aeruginosa*

We then quantified the transcription of genes encoding the efflux pump proteins MexA, MexC, MexE, and MexX. The median results of gene-expression analyses during eight passages of the six lineages selected by ciprofloxacin and levofloxacin are shown in Tables 3 and 4, respectively. The results showed that overexpression of MexC was the most important mechanism contributing to FQ resistance, whereas MexE and MexX played supplementary roles (Fig. 1). For levofloxacin selection, the first significant overexpression of these three genes occurred at 0.5 mg/L (1MIC), whereas this occurred at 0.25 mg/L (0.5MIC) for ciprofloxacin selection. Moreover, the second significant overexpression was observed at 1 mg/L (2MIC) for MexC in case of ciprofloxacin selection and at 2 mg/L (4MIC) in case of levofloxacin selection. These results suggested that ciprofloxacin showed a stronger effect on inducing resistance in *P. aeruginosa* relative to that observed for levofloxacin and within the clinical breakpoint recommended by the CLSI (initial breakpoint: ciprofloxacin ≤0.5 mg/L and levofloxacin ≤1 mg/L; intermediate breakpoint: ciprofloxacin = 1 mg/L and levofloxacin = 2 mg/L).

**Table 3:** Expression of efflux pump genes in evolved, resistant *P. aeruginosa,* selected by ciprofloxacin^a^

Passage	*mexA*	*mexC*	*mexE*	*mexX*
0. (0MIC)	1.0000	1.0000	1.0000	1.0000
1. (1/2MIC)	1.3075	9.0280	11.9735	2.3705
2. (1MIC)	0.6615	9.9210	10.1515	1.3515
3. (2MIC)	1.0715	144.4805	6.5980	2.1285
4. (4MIC)	0.8040	11.4565	6.3370	1.4735
5. (8MIC)	0.6125	86.9080	5.0330	1.7635
6. (16MIC)	0.8320	195.6205	8.3245	3.5535
7. (32MIC)	0.6140	132.7665	7.0495	3.5345
8. (64MIC)	0.7175	112.6305	10.0960	4.5755

^a^Relative expression levels represent the median of the six lineages

**Table 4:** Expression of efflux pump genes in evolved, resistant *P. aeruginosa,* selected by levofloxacin^a^

Passage	*mexA*	*mexC*	*mexE*	*mexX*
0. (0MIC)	1.0000	1.0000	1.0000	1.0000
1. (1/2MIC)	0.6700	2.6295	1.4200	1.3850
2. (1MIC)	1.2310	8.6650	5.4625	2.2925
3. (2MIC)	0.9325	16.2745	8.3645	1.2030
4. (4MIC)	0.8605	152.1040	2.6225	3.0995
5. (8MIC)	1.2310	52.7525	12.0805	1.3095
6. (16MIC)	1.0385	57.6390	15.4695	2.0900
7. (32MIC)	1.4255	145.8855	10.5065	4.6735
8. (64MIC)	1.5140	74.2490	12.8310	7.5555

^a^Relative expression levels represent the median of the six lineages

### Correlation between each mutation and efflux pump gene expression

**Fig. 2:**
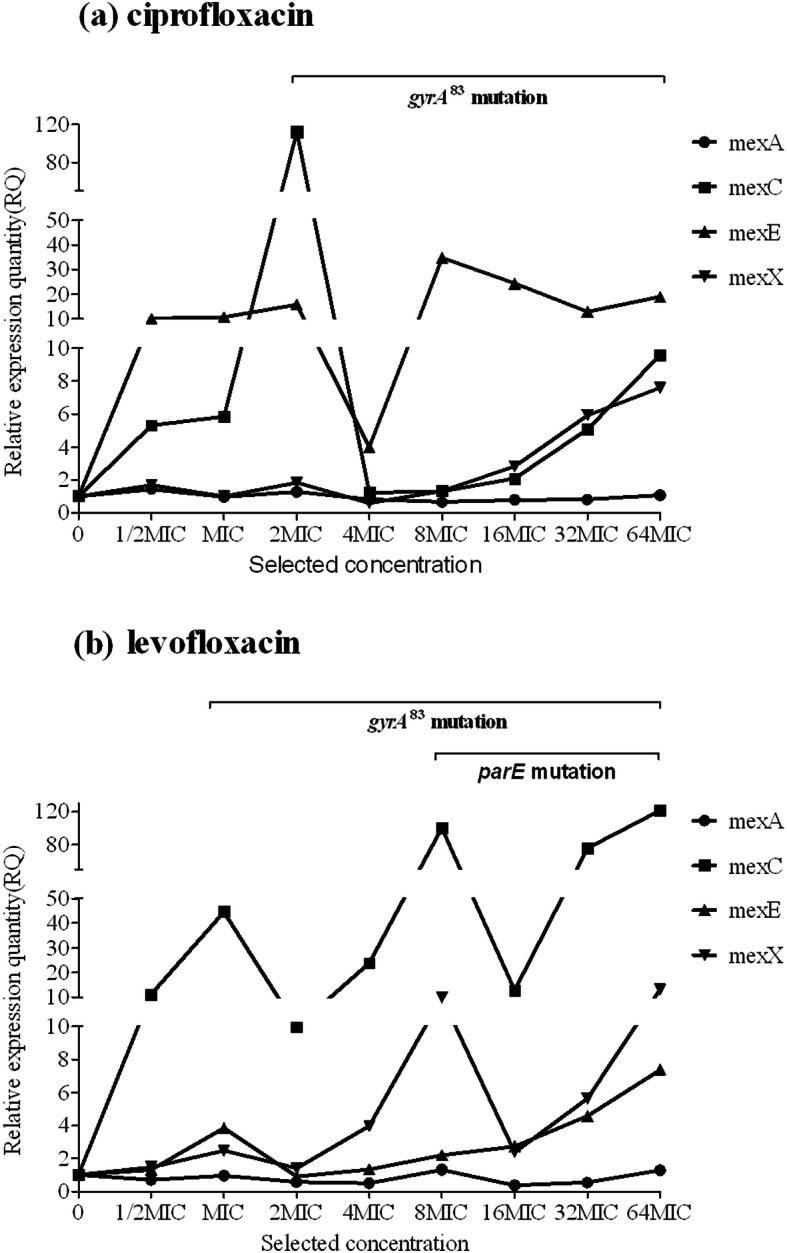
Correlation between each mutation and efflux pump gene expression in lineage 1 of evolved, resistant *P. aeruginosa*

**Fig. 3:**
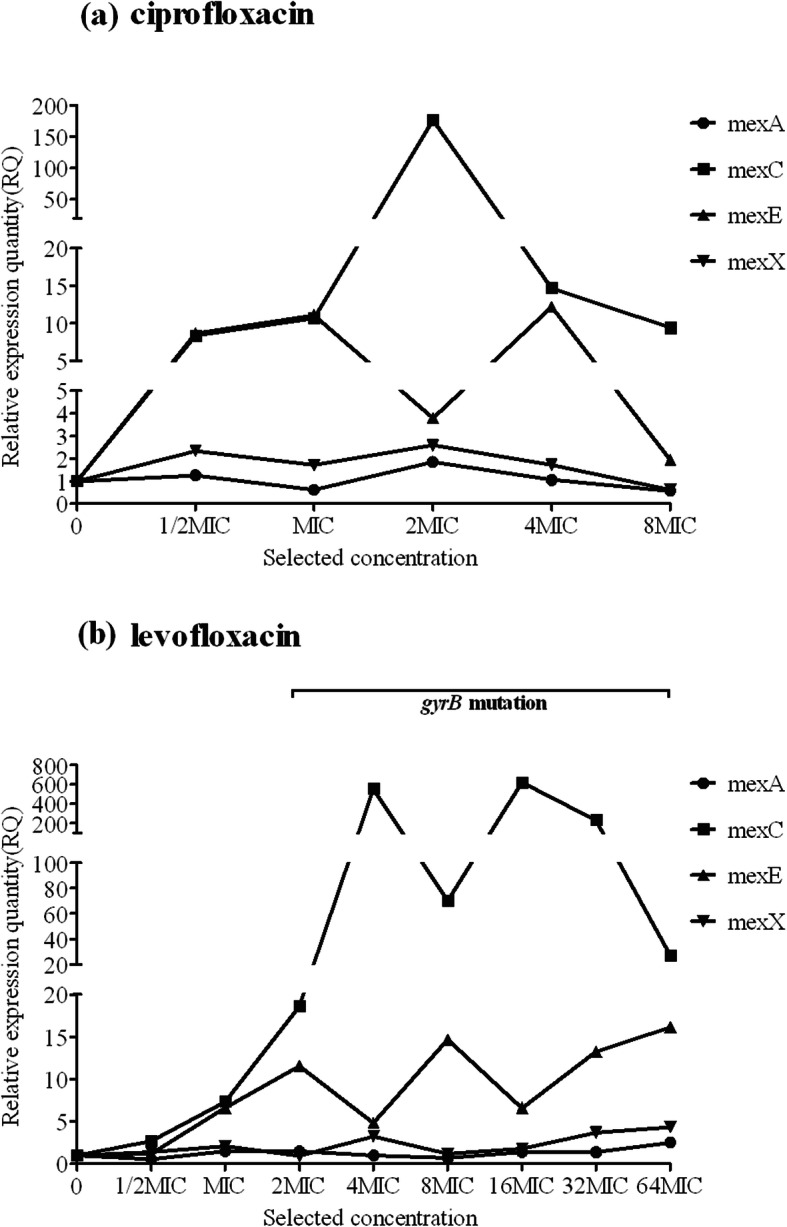
Correlation between each mutation and efflux pump gene expression in lineage 5 of evolved, resistant *P. aeruginosa*

We identified several significant correlations between QRDR mutations and efflux pump expression. First, the presence of the *gyrA*^83^ mutation at lower selected concentrations, such as L1 for ciprofloxacin and L1 and L4 for levofloxacin (Fig. 2), correlated with downregulated expression of the four different efflux pump genes, whereas the *gyrA*^87^mutation did not have the same effect. Second, the presence of the *parE*^457^ mutation in L1 for levofloxacin was concomitant with downregulated expression of *mexC* and *mexE*. Third, levofloxacin selection of L3, L5, and L6 before the appearance of the *gyrA*^83^ mutation or with the *gyrB*^466^ mutation alone (or with no mutation, as observed in L5 for ciprofloxacin selection) (Fig. 3) resulted in synergistic regulation of *mexC* and *mexE* expression (upregulated *mexC* expression corresponded to downregulated *mexE* expression).

## Discussion

Microbial evolution experiments are powerful tools for investigating bacterial adaptation to antibiotic stress under controlled laboratory conditions and are commonly used to assess the causes and dynamics of the evolution of antibiotic resistance in bacteria [7]. According to our previous experiments in a lung infection model in rats, we were only able to find plasmid-mediated resistance rather than QRDR mutations, which were highly detected in clinical studies [8]. However, in the present study, we developed *P. aeruginosa* resistance in liquid medium in vitro in the presence of sub-lethal concentrations of antibiotics (0.5MIC of the parental strain, ATCC27853), increasing up to 64MIC. Although the detection method was the same, the results were completely different from those obtained by step-wise selection of resistant mutants in animal models, and were similar to clinical studies. Here, we were able to effectively block communication between *P. aeruginosa* and the environment, thereby allowing a focus on the development of *P. aeruginosa* resistance under increasing antibiotic pressure. The results identified chromosome-mediated QRDR mutations, which were highly similar to those detected in clinical studies, thus providing a reference for clinical treatment. Furthermore, previous studies used agar plates as the medium for the bacterial growth and chose a single colony for subsequent passages and sequencing [9]; however, this method can exclude emerging mutations existing in minor colonies [10]. Therefore, in the present study, we incubated bacteria in liquid medium and extracted DNA and RNA from the collected media containing the resistant bacteria obtained from various resistance mechanisms, as previously demonstrated during identification of progressively increasing numbers of QRDR mutations in *Streptococcus pneumoniae* [4]. This method allowed detection of low-frequency mutations during the evaluation (e.g., *parE*^45 7^[S457C] and *gyrB*^466^ [S466F]).

We identified five different mutations in QRDRs, of which three (*gyrA*^83^ [T83I], *gyrA*^87^ [D87N], and *gyrB*^466^ [S466F]) were consistent with previous clinical detection of *P. aeruginosa*-resistant populations [3, 11]. Additionally, we detected replacement of serine at position 457 by cysteine in *parE*, which was similar to clinical results showing an altered arginine residue [12]. However, we did not identify a *parC* mutation unlike previous clinical results from sequencing both clinical isolates and laboratory derived mutations of *Mycoplasma bovis*, which showed different abilities in the development of resistance [13]. We speculate that the *parC* mutation could be related to the drug combination or hospital environment associated with its identification. Here, we used an in vitro model to investigate the mechanisms of *P. aeruginosa* resistance to ciprofloxacin and levofloxacin in order to overcome uncertainties related to clinical treatment. Moreover, in vitro analysis aimed to determine the resistance mechanisms of *P. aeruginosa* to ciprofloxacin and levofloxacin in the absence of an immune system. Therefore, the results might differ from those determined in animal models or humans. Furthermore, the methods used in this study could be repeated on a cohort of clinical isolates.

Our results showed that the evolutionary trajectories of FQ resistance are more complicated than previously described. As per previous studies, ciprofloxacin and levofloxacin develop resistance through different mechanisms. In this study, mutation in *gyrA*^83^ occurred in two lineages in case of ciprofloxacin and in five lineages in case of levofloxacin. However, there were two kinds of *gyrA*^87^ mutations only under ciprofloxacin pressure, and the three different *gyrA* mutations did not appear at the same time. Moreover, the mutation identified in *parE* was found in only one lineage that evolved under levofloxacin pressure together with *gyrA*^83^ mutation. The mutation in *gyrB* was consistently found in combination with the *gyrA* mutations but disappeared during the evolutionary process in L2 during ciprofloxacin selection, and in L3 and L6 during levofloxacin. These phenomena observed in our study are similar to clinical results showing *gyrA* mutation as the main resistance mechanism in *P. aeruginosa*, whereas the *gyrB* mutation could reinforce its resistance ability [11, 14]. Furthermore, these studies demonstrated recovery from *gyrB* mutation at high drug concentrations, as well as rare occurrences of *ParE* mutations [11, 14]. In the present study, we showed that the main mechanism contributing to resistance in *P. aeruginosa* involved the expression of efflux pump genes, specifically overexpression of MexCD-OprJ and upregulation of MexEF-OprN and MexXY-OprM. These results provided in vitro confirmation of previous clinical reports [12, 15, 16].

Further analysis of the lineages from the perspective of the two different mechanisms enabled identification of a relationship between gene mutations and overexpression of efflux pump genes, with *gyrA*^83^ mutation correlating with downregulated expression of *mexC*, whereas this was not observed with *gyrA*^87^ and *gyrB*^466^.

In summary, we showed that ciprofloxacin had a stronger ability to kill *P. aeruginosa*, whereas specific mutations and overexpression of efflux pumps might render the bacteria more susceptible to resistance. These findings indicate that although ciprofloxacin and levofloxacin are both FQs, they demonstrate different abilities in their bactericidal action and induction of resistance.

## Supplementary information

**Additional file 1 **: **Table S1**. Primers for QRDR amplified in this study. **Table S2**. Primers used for real-time PCR in this study [3].

## Data Availability

The datasets used and/or analyzed in this study are available from the corresponding author upon reasonable request.

## Abbreviations

CLSI: Clinical & Laboratory Standards Institute
FQ: Fluoroquinolone
MHB: Mueller−Hinton broth
MIC: Minimum inhibitory concentration
PCR: Polymerase chain reaction
QRDR: Quinolone-resistance determining region
