# Supplementary material for: Development of in vitro resistance to fluoroquinolones in Pseudomonas aeruginosa
Source: Antimicrob Resist Infect Control. 2020 Aug 5;9:124. doi: 10.1186/s13756-020-00793-8 (PMC7405379; doi:10.1186/s13756-020-00793-8)
Supplement: Supplementary file 1 — Additional file 1 : Table S1. Primers for QRDR amplified in this study. Table S2. Primers used for real-time PCR in this study [3]. [file 13756_2020_793_MOESM1_ESM.doc]

Table1.Primers for QRDR amplified in this study

| gene | primer | reference |
| --- | --- | --- |
| *gyrA* | F 5’-AGTCCTATCTCGACTACGCGAT-3’ |  |
|  | R 5’-AGTCGACGGTTTCCTTTTCCAG-3’ |  |
| *gyrB* | F 5' -GAACTGTACATCGTGGAGGGTGA-3' |  |
|  | R 5' -ACCCCGCGATACTCGTTGAC-3' |  |
| *parC* | F 5' -CGAGCAGGCCTATCTGAACTAT-3' |  |
|  | R 5' -GAAGGACTTGGGATCGTCCGGA-3' |  |
| *parE* | F 5' -AGGACGCCTTCAGCCTGTG-3' |  |
|  | R 5' -GGAAATGGCGGACGAACAG-3' |  |

Table2.Primers used for real-time PCR in this study[3]

| gene | primer |
| --- | --- |
| *rpoD* | F 5’-CGCAACAGCAATCTCGTCTGAAA-3’ |
|  | R 5’-GCGGATGATGTCTTCCACCTGTT-3’ |
| *mexA* | F 5’-GGCGACAACGCGGCGAAGG-3’ |
|  | R 5’-CCTTCTGCTTGACGCCTTCCTGC-3’ |
| *mexC* | F 5’-GCAATAGGAAGGATCGGGGCGTTGG-3’ |
|  | R 5’-CCTCCACCGGCAACACCATTTCG-3’ |
| *mexE* | F 5’-TCATCCCACTTCTCCTGGCGCTACC-3’ |
|  | R 5’-CGTCCCACTCGTTCAGCGGTTGTTCGATG-3’ |
| *mexX* | F 5’-AATCGAGGGACACCCATGCACATCC-3’ |
|  | R 5’-CCCAGCAGGAATAGGGCGACCAG-3’ |
